# Morphological Bases of Suppressive and Facilitative Spatial Summation in the Striate Cortex of the Cat

**DOI:** 10.1371/journal.pone.0015025

**Published:** 2010-11-29

**Authors:** Xue-Mei Song, Ye Wang, Zhao Zhu, Chao-Yi Li

**Affiliations:** 1 Shanghai Institutes of Biological Sciences, Chinese Academy of Sciences, Shanghai, China; 2 Key Laboratory for Neuroinformatics, Ministry of Education of China, University of Electronic Sciences and Technology, Chengdu, China; Dalhousie University, Canada

## Abstract

In V1 of cats and monkeys, activity of neurons evoked by stimuli within the receptive field can be modulated by stimuli in the extra-receptive field (ERF). This modulating effect can be suppressive (S-ERF) or facilitatory (F-ERF) and plays different roles in visual information processing. Little is known about the cellular bases underlying the different types of ERF modulating effects. Here, we focus on the morphological differences between the S-ERF and F-ERF neurons. Single unit activities were recorded from V1 of the cat. The ERF properties of each neuron were assessed by area-response functions using sinusoidal grating stimuli. On completion of the functional tests, the cells were injected intracellularly with biocytin. The labeled cells were reconstructed and morphologically characterized in terms of the ERF modulation effects. We show that the vast majority of S-ERF neurons and F-ERF neurons are pyramidal cells and that the two types of cells clearly differ in the size of the soma, in complexity of dendrite branching, in spine size and density, and in the range of innervations of the axon collaterals. We propose that different pyramidal cell phenotypes reflect a high degree of specificity of neuronal connections associated with different types of spatial modulation.

## Introduction

Each neuron in area V1 responds only to visual stimuli located within a limited area of visual space, an area referred to as the classical receptive field (CRF). The CRF of V1 neurons was thought to play a role in coding information about simple visual features, such as the orientation and spatial frequency of luminance contrasts. Recent studies have shown that the extensive field beyond the CRF of V1 cells – the extra-receptive field (ERF) – though alone unresponsive to visual stimuli,can modulate the response elicited by stimuli located inside the CRF [1]–[16]. This modulatory effect can be suppressive (S-ERF) or facilitatory (F-ERF), and its extent is about three to five times larger in diameter than the CRF [3], [4], [17], [18], [19], [20]. As this modulation is azlso feature dependent, V1 cells signal not only the local features within the CRF, but also convey information about the context of visual features over an extensive area [1]–[9], [21], [22].

It was reported that the cells with F-ERF respond to homogeneity or similarity of texture features, whereas the cells with S-ERF respond to heterogeneity or differences in the visual contexture [4]–[6], [23]. The opponent effects of the two types of ERFs combined together enable cortical neurons to encode complex visual textures in the natural scene, and this has been interpreted to be the neural substrate of figure-ground segregation [7], [24] as well as a variety of visual perceptions [2], [10], [25], [26].

The aim of the present study was to characterize neurons in terms of surround modulation, i.e., suppression or facilitation of the ERF, and to correlate the different modulation effects with the morphological features of the associated cells. Therefore, on conclusion of functional tests, the functionally identified cells were injected intracellularly with biocytin [27]. The labeled cells were reconstructed and morphologically characterized. The axon and dendritic morphology of the labeled cells were compared with their functional properties in terms of their surround modulation effect. Set against these observations, we tried to explain the functional differentiation in surround modulation in terms of the neuron's morphological differences.

## Results

### Determination of CRF and ERF Properties

**Figure 1:**
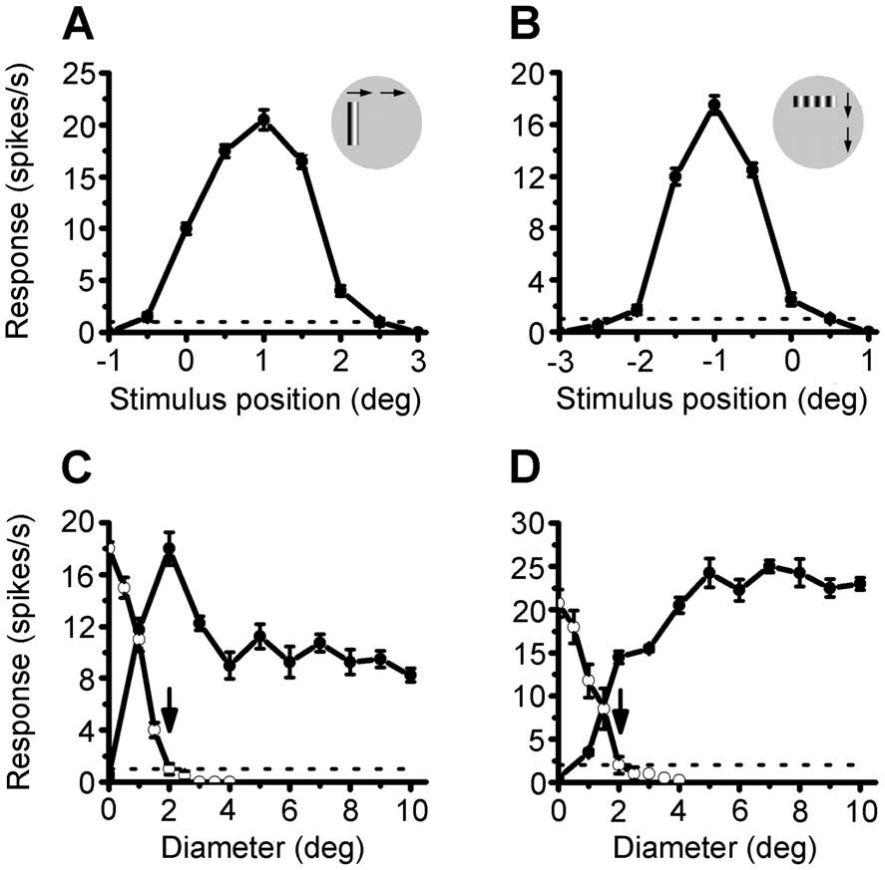
Determination of CRF Size and ERF Properties. (A and B) Response profiles used to determine the CRF size and center location. A narrow sine-wave grating patch was placed in a random sequence at successive positions along the axes perpendicular (inset in A) or parallel (inset in B) to the optimal orientation of the cell, and response of the cell was measured at different stimulus positions to the drift of the grating patch. The peak of the response profiles for both axes was defined as the center of the CRF. The response field above the spontaneous level (dashed line) is defined as the length and width of CRF. Zero on the abscissa represents the location of the central area of retina. (Cell no. 27 in Table 1). (C) Occlusion test and spatial summation test of a neuron with S-ERF (the same cell as in A, *SI* = −0.58). The descending line on the left shows the result of occlusion test. The diameter of the mask at which the response decreased to the spontaneous level (dashed line) was defined as the size of CRF (arrow). The other line shows the spatial summation test of the cell. Maximum response was found for a stimulus diameter corresponding to the size of the CRF. The stimulus diameter by which the response decreased to a minimum level (4° diameter) indicates the size of the S-ERF. (D) Occlusion test and spatial summation test of a neuron with F-ERF (Cell no. 11 in Table 1, *SI* = 0.33). The descending line shows the result of occlusion test, and the other line shows spatial summation test for the cell. Response of the cell increased progressively with increasing stimulus size both within and beyond the CRF (arrow). A plateau was reached at a diameter (about 5°) representing the size of the F-ERF. Error bars indicate SEM.

Sinusoidal grating patterns drifting at the optimal orientation and spatiotemporal frequency were used to determine the center of CRF and the properties of ERF for the neurons. We first located the center of CRF by placing a narrow sine-wave grating patch (40% contrast) at successive positions (in a random sequence) along the axes perpendicular or parallel to the optimal orientation of the cell and measuring the response to its drift. The peak of the response profiles for both axes was defined as the center of the CRF (Figure 1A and B). All the recorded cells had CRFs centered within 10° of the visual axis. We then measured the CRF diameter by performing an occlusion test [4], [28], in which a mask of circular blank patch, concentric with the CRF, was gradually increased in size on a background drifting grating. The size of the mask at which the neuronal response decreased to the spontaneous level was defined as the diameter of the CRF. The results of occlusion test for two different cells are shown in Figure 1C and D by the descending lines and the CRF sizes thus measured are indicated by the arrows.

The ERF properties of neurons were assessed by measuring the neuron's response as a function of stimulus area [4], [28]. Circular drifting sinusoidal grating patches of different diameters were used as the stimulus. The gratings were presented at the optimal orientation and spatiotemporal frequency in a random sequence; each patch size was presented for 5–10 cycles of the grating drift, and standard errors were calculated for 3–10 repeats. Outside the grating patches, the screen (40°×30°) was kept at a mean luminance of 10 cd/m^2^. The spatial summation curve thus obtained reflects the influence of the surrounding area on the CRF responses. We identified two classes of ERFs based on the shape of the spatial summation curve. The first class was the suppressive ERF illustrated by the example shown in Figure 1C. Maximum neuronal response was found for a stimulus diameter corresponding to the size of the CRF (indicated by the arrow in Figure 1C), and the response decreased with increasing grating size beyond the CRF. The stimulus diameter by which the response decreased to a minimum level indicates the size of the S-ERF. The second class was the facilitatory ERF, which showed continuously increasing neuronal response even as the stimulus size increased beyond the CRF (arrow in Figure 1D). A plateau (maximum) was reached at a diameter, which represents the size of the F-ERF. For most of the striate cortical cells, the size of the ERF varied between 3–5 fold the CRF size.

To quantitatively estimate the ERF properties of the cortical neurons, we defined a summation index (*SI*) by *SI = (R_ff_ -R_crf_)/(R_max_ - R_spt_)*_,_ where *R_ff_* represents the response amplitude under full field stimulation (the grating at 5x CRF size stimulation), R_crf_ represents the response to CRF stimulation alone, *R_max_* is the maximum response of the cell, and *R_spt_* represents the spontaneous discharge rate. For neurons with S-ERF, *SI* is <0 and >−1, forF-ERF neurons, *SI* is >0 and <1. For clarity of morphological analysis, we used to label principally the typical facilitatory and suppressive ERF neurons, i.e., the neurons with *SI* 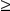
0.2 or *SI* 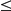 −0.4.

### Cellular morphology of F- ERF and S-ERF neurons

We have investigated a total of 44 V1 cells for which both functional analyses and intracellular staining were successfully achieved. Of the sample, 22 were located in the superficial layers (layers II/III) where 13 were identified as S-ERF neurons and 9 as F-ERF neurons. Of the remaining 22 located in the deeper layers (layers IV, V and VI), 12 were F-ERF neurons and 10 were S-ERF neurons. Morphologically, 19 out of 21 of the F-ERF neurons and 19 of the 23 S-ERF neurons were pyramidal cells, only 2 out of 21 of the F-ERF neurons were spiny stellate cells and 4 out of 23 from the S-ERF neurons were smooth interneurons. Table 1 summarizes the layer distribution, CRF size, summation index, morphological feature and the simple/complex categorization of all these neurons. Classifying simple and complex cells is based on the relative modulation in the responses to drifting sinusoidal gratings (the ratio of the first harmonic to the mean firing rate, after subtracting the average spontaneous rate) [29].

**Table 1:** Summarization of layer distribution, morphology and functional properties of the sample of neurons.

F-ERF neurons (n = 21)						I-ERF neurons (n = 23)					
Cell no.	Morp	Layer	CRF	SI	S/C	Cell no.	Morp	Layer	CRF	SI	S/C
1	▴	II-III	2.0	0.28	S	22	▴	II–III	4.0	−0.88	C
2	▴	II–III	4.0	0.48	C	23	▴	II–III	2.0	−0.24	S
3	▴	II -III	5.0	0.38	C	24	▴	II–III	4.0	−0.94	C
4	▴	II–III	4.0	0.27	C	25	▴	II–III	2.0	−0.57	S
5	▴	II–III	2.0	0.25	S	26	▴	II–III	2.0	−0.60	C
6	▴	II–III	2.5	0.67	S	27	▴	II–III	2.0	−0.58	S
7	▴	II–III	2.5	0.39	C	28	▴	II–III	4.0	−0.46	C
8	▴	II–III	3.0	0.47	C	29	▴	II–III	3.5	−0.28	C
9	▴	II–III	4.0	0.28	C	30	▴	II–III	4.0	−0.82	S
10	▴	IV	4.0	0.53	S	31	▴	II–III	4.0	−0.77	C
11	▴	IV	2.0	0.33	S	32	▴	II–III	4.0	−0.53	C
12	▴	IV	4.0	0.32	S	33	▴	IV	2.0	−0.59	C
13	▴	IV	2.0	0.39	S	34	▴	IV	1.0	−0.80	C
14	▴	IV	5.0	0.34	S	35	▴	V	2.0	−0.62	S
15	▴	IV	4.0	0.48	S	36	▴	V	4.0	−0.63	C
16	▴	IV	4.0	0.71	S	37	▴	V	2.0	−0.55	C
17	▴	VI	4.0	0.23	C	38	▴	V	3.0	−0.28	C
18	▴	VI	4.0	0.32	S	39	▴	V	2.0	−0.56	S
19	▴	VI	5.0	0.50	S	40	▴	VI	2.0	−0.71	C
20	★	IV	2.0	0.35	S	41	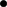	II–III	4.0	−0.52	C
21	★	IV	2.0	0.60	S	42	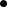	II–III	2.0	−0.50	S
						43	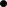	IV	1.0	−0.85	C
						44	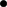	V	3.0	−0.28	C

**CRF**: diameter of CRF; **SI**: value of summation index; **S/C**: simple vs complex cell. ▴ pyramidal cell, ★ spiny stellates cell, 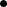 smooth dendritic neuron.

### Comparison of dendritic morphologies between F-ERF and S-ERF neurons

**Figure 2:**
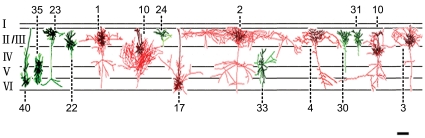
Morphological features and laminar distribution of 15 injected neurons conformed to the common cortical template. The data were collected from different animals. For better illustration, two colors (red and green) were used. For F-ERF neurons, axons are shown in bright red and dendrites are shown in dark red. For S-ERF neurons, axons are shown in bright green, and dendrites are shown in dark green. Neurons with F-ERF have widely distributed axon collaterals. Neurons with S-ERF have fewer dendrite branches, and their axon collaterals are close to the cell body. Roman numerals on the left indicate the cortical layers, and Arabic numerals denote the cell numbers which are equivalent to the cell numbers in Table 1. Scale bar: 300 µm (not adjusted for shrinkage).

**Figure 3:**
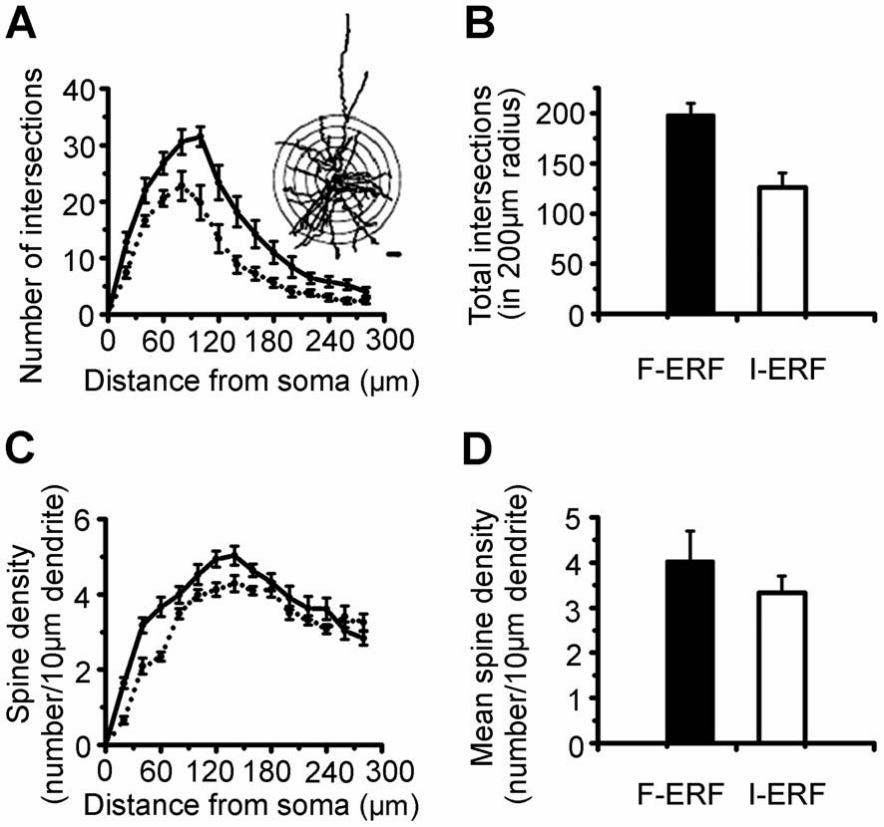
Plot of Sholl analyses of dendrite bifurcations and spine density of F-ERF and S-ERF neurons. A sample of 10 pyramidal cells (5 F-ERF neurons and 5 S-ERF neurons) were analyzed. In A and C, the dark curve represents the mean result from the F-ERF neurons, and the dashed curve, the mean result for the S-ERF neurons. Error bars represent ± SEM. (A) Dendrite Sholl analysis of the two types of neurons. Inset illustrates the method of Sholl analysis, Bar = 20 µm. Number of Sholl intersections along the dendritic trees at all distances from the soma revealed statistically significant differences between the F-ERF and S-ERF neurons (t-test, P<0.05). (B) Comparison of total number of Sholl intersections between the F-ERF and S-ERF cells. Number of intersections was counted in 200 µm radius from the soma along the dendritic tree. The mean intersections for F-ERF cell group (n = 5) is 198.0
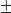
11.9 and that for the S-ERF cell group (n = 5) is 126.2
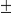
14.2. The difference is significant (P<0.05, t-test). (C) Plots of spine densities as a function of distance from the soma. Number of spines was calculated per 20 µm dendrite. The dendrites of F-ERF neurons have a higher spine density than S-ERF neurons, especially at the proximal segments of dendrites (20, 40, 60 µm levels from cell body, t-test, P<0.01). (D) Comparison of mean spine density between the F-ERF and S-ERF cells. Spine density (number of spines/10 µm dendrite) was averaged for the sample of cells in 200 µm radius from the soma. The mean spine density for the F-ERF neuron group is 4.03
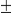
0.67 and that for the S-ERF neuron group is 3.33
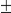
0.37. The difference is not significant (P>0.05, t-test). All values were obtained after correction for shrinkage.

The dendritic and axon morphologies and the distribution by layer of 15 injected cells (7 F-ERF, 8 S-ERF) are illustrated in Figure 2. Numerals in this figure denote the cell numbers equivalent to the cell numbers listed in Table 1. To aid visualization, axons of F-ERF neuron are depicted in bright red and their dendrites in dark red; axons of S-ERF neuron are depicted in bright green and their dendrites in dark green. Horizontal lines indicate the approximate cortical layers where the cells were located. Although at first glance the dendritic morphology of F-ERF and S-ERF neurons seemed to be not significantly different, quantitative analysis of 10 pyramidal cells (5 F-ERF neurons and 5 S-ERF neurons) revealed that the F-ERF neurons have an apparently more complex dendritic arborization than the S-ERF neurons. The complexity of dendritic branching of the two types of neurons was assessed using the Sholl analysis [30]. In Figure 3A the number of dendrite segments was counted by intersections crossing each 20-µm-radius ring progressively more distal to the soma. When the entire (apical and basal) dendritic tree was analyzed as a whole, there were apparently more intersections (indicating more bifurcations on the dendrites) in the F-ERF neurons than in S-ERF neurons for all radii between 20 µm and 260 µm (P<0.01 from 20 µm to 180 µm radii, and P<0.05 for all radii between 180 µm and 260 µm). The peak dendritic field complexity was located between 60 and 80 µm from the cell body for S-ERF neurons, and 80 and 100 µm for F-ERF neurons, beyond which the number of dendritic branches decreased. Figure 3B shows a comparison of the total number of Sholl intersections between the F-ERF and S-ERF cells. Number of intersections was counted in 200 µm radius from the soma along the dendritic tree. The mean number of intersections for the five F-ERF neurons is 198.0
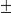
11.9 and that for the five S-ERF neurons is 126.2
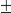
14.2. The difference is significant (P<0.05, t-test).

### Comparison of spine density and spine-head areas

In addition, we compared the spine density of dendrites between the two types of neurons. In Figure 3C spine densities of the same sample of cells are plotted as a function of the distance from the cell body. The results reveal that spine density varies as a function of the distance from the cell body, reaching a peak at ∼120–160 µm from the soma. It is clear that the dendrites of F-ERF neurons have higher spine density than S-ERF neurons, especially at the proximal segments of dendrites (20, 40, 60 µm levels from cell body, t-test, P<0.01). Figure 3D shows a comparison of mean spine density between the F-ERF and S-ERF cells. Here spine density (number of spines/10 µm dendrite) was averaged in 200 µm radius from the soma. The mean value for F-ERF neuron group is 4.03
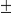
0.67, and that for the S-ERF neuron group is 3.33
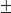
0.37. The difference is not significant (P>0.05, t-test).

**Figure 4:**
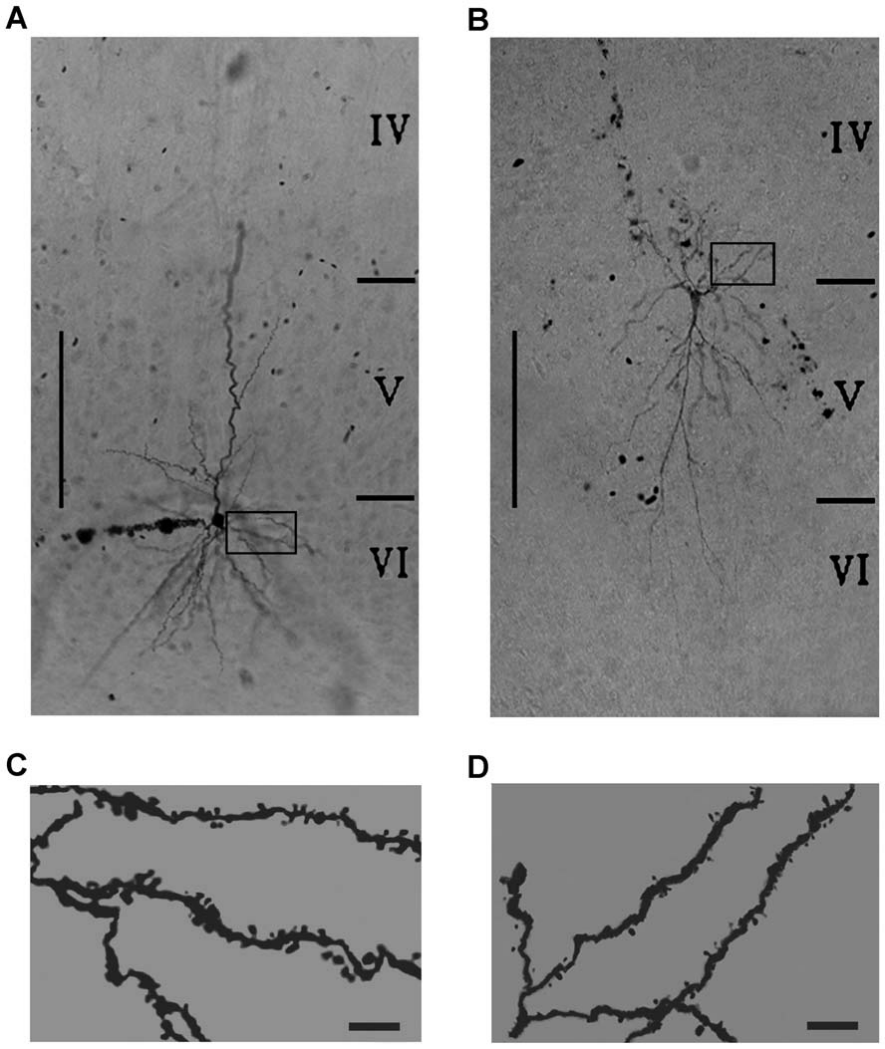
Differences in dendrite spines between F-ERF and S-ERF pyramidal neurons identified with DAB. (A) Image of an F-ERF neuron (SI = 0.23) in layer VI (number 17 in Table 1). (B) Image of an S-ERF neuron (SI = −0.62), which was an inverted pyramidal cell in layer V (number 35 in Table 1). (C) High magnification of a fraction of basal dendrites of the neuron in A (indicated by the rectangle in A) showing that the majority of spines for F-ERF neurons have a thicker and shorter neck with a larger head. (D) High magnification of dendrites of the neuron in B (indicated by the rectangle in B) illustrating that the majority of spines for S-ERF neurons are characterized by a thinner and longer neck with a small head. Roman numerals on the right in A and B indicate the cortical layers. Scale bar in A and B: 300 µm, and in C and D: 5 µm (not adjusted for shrinkage).

**Figure 5 pone-0015025-g005:**
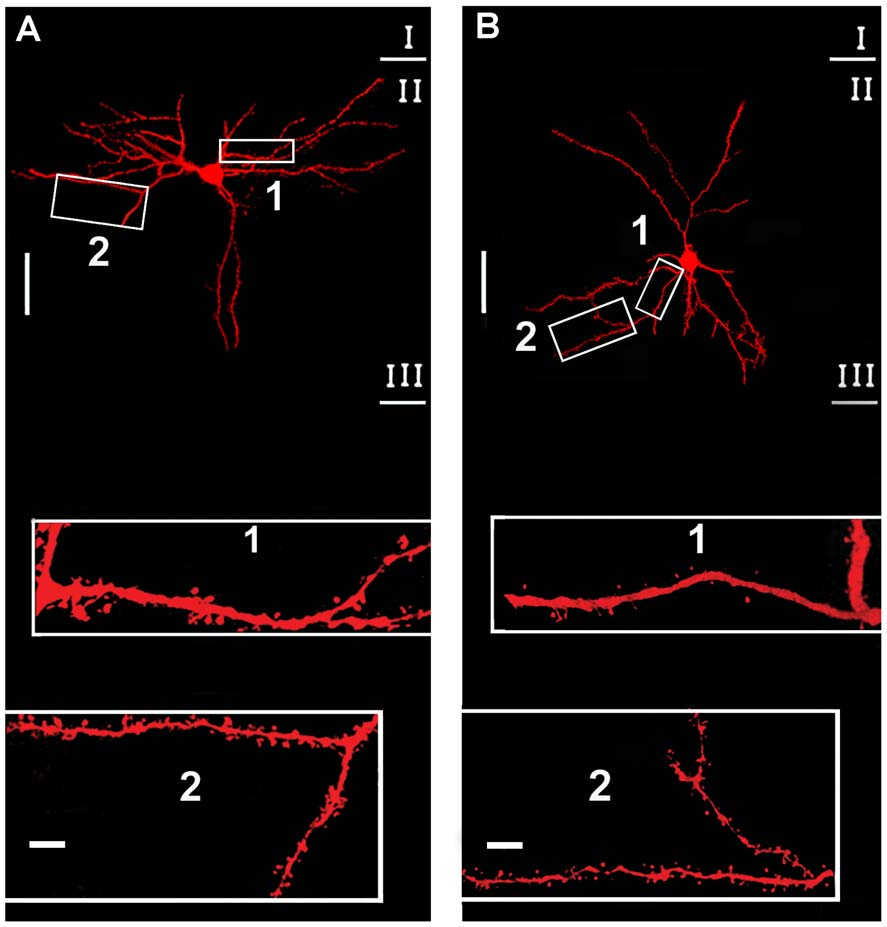
Differences in dendrite spines between F-ERF and S-ERF pyramidal neurons identified with streptavidin-Texas Red. (A) Image of an F-ERF neuron in layer II/III (SI = 0.25, cell No. 5 in Table 1). (B) Image of an S-ERF neuron in layer II/III (SI = −0.57, No. 25 in Table 1). The magnified images in squares 1 and 2 illustrate the differences in spine morphology between the F-ERF (A) and the S-ERF neurons (B). Scale bar in A and B:100 µm, and in insets: 5 µm.

**Figure 6:**
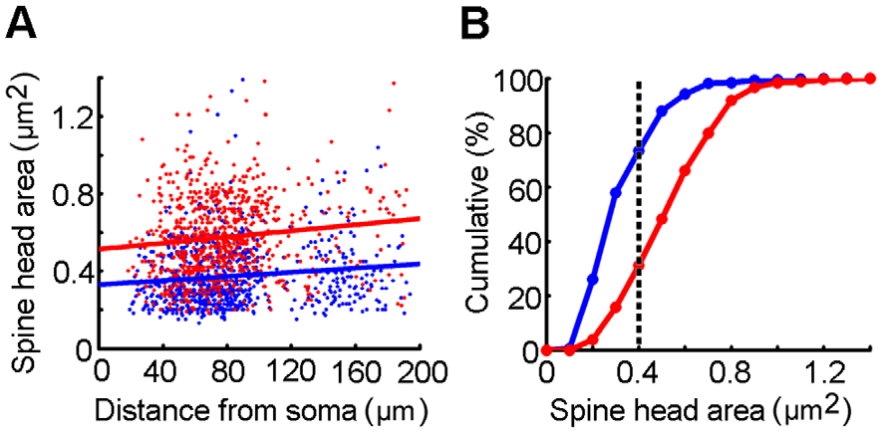
A comparison of spine head area between F-ERF and S-ERF pyramidal neurons. Eight pyramidal neurons (4 F-ERF neurons, 4 S-ERF neurons) identified with DAB were analyzed and a total of 1600 spine heads were measured. (A) Distribution of spine-head areas of the F-ERF and S-ERF neurons at different distance from the soma. Red dots represent the data from the F-ERF neurons, and blue dots, the data from S-ERF neurons. Red line is the regression line of the data points of the F-ERF neurons (r = 0.1239, P<0.001), and blue line, the regression line of the data points of the S-ERF neurons (r = 0.1249, P<0.001). No correlation was found between spine head area and distance to soma. (B) The cumulative curve of spine head area. Red dots displayed the results for the F-ERF neurons and blue dots displayed the results from S-ERF neurons. The y-axis values represent cumulative frequencies in % and the x-axis values, the area of spine heads. The vertical dotted line on the x-axis indicates that, for F-ERF neurons, about 75% of the spines had heads >0.4 µm^2^ compared to S-ERF neurons which had about 75% of spine heads <0.4 µm^2^ (not adjusted for shrinkage).

Furthermore, the shape and size of dendritic spines also differ between F-ERF and S-ERF cells. A visual comparison between the two types of spines is shown in Figure 4. The neuron in Figure 4A was an F-ERF pyramidal cell and that in Figure 4B an S-ERF pyramidal cell, both were labeled with avidin-HRP by the intracellular injection of biocytin. The high-power photomicrographs of a fraction of their basal dendrites (indicated by the squares) illustrate that the predominant types of spines for F-ERF cells (Figure 4C) have a thicker and shorter neck with a larger head, in contrast, most of the spines for S-ERF neurons (Figure 4D) are characterized by a thinner and longer neck expanding into a small head. Similar differences can be seen in Figure 5 where an injected F-ERF pyramidal cell (Figure 5A) and an S-ERF pyramidal cell (Figure 5B), both were in layer II-III, were identified with fluorescent dye (streptavidin Texas Red). To quantitatively characterize the morphology of the spines, we made measurements of the spine-head areas for 8 pyramidal cells (4 F-ERF neurons, and 4 S-ERF neurons). For each neuron, ten dendrite segments were examined and twenty spines were sampled for each segment. The analysis of dendritic spines was limited to the segments up to 200 µm from the soma. The spine-head areas of a total of 1600 spine heads were measured and their distribution at different distances from the soma is shown in Figure 6A, where the red dots represent the distribution of spine-head areas of F-ERF neurons, and the blue dots represent the distribution of spine-head areas of S-ERF neurons. The red and blue lines represent, respectively, the regression line of the data points of the F-ERF neurons (r = 0.1239, P<0.001, n = 800) and that of the S-ERF neurons (r = 0.1249, P<0.001, n = 800). No correlation was found between spine head area and the distance from the soma. Lack of correlation between spine-head volume and distance to soma was also reported by Arellano et al [31]. Figure 6B shows the distribution cumulative frequency of spine head area along the dendrite arbors for the F-ERF neurons (red dots) and the S-ERF neurons (blue dots). The y-axis values represent cumulative frequencies in %, and the x-axis values, the area of spine heads. The mean spine-head area for the S-ERF neurons (n = 4) was 0.37 µm^2^ (SD = 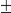
0.17 µm^2^) and that for the F-ERF neurons (n = 4) was 0.57 µm^2^ (SD = 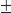
0.21 µm^2^). The vertical dotted line on the x-axis indicates that, for F-ERF neurons about 75% of the spines had heads >0.4 µm^2^ compared to S-ERF neurons which had about 75% of the spine heads <0.4 µm^2^.

**Figure 7:**
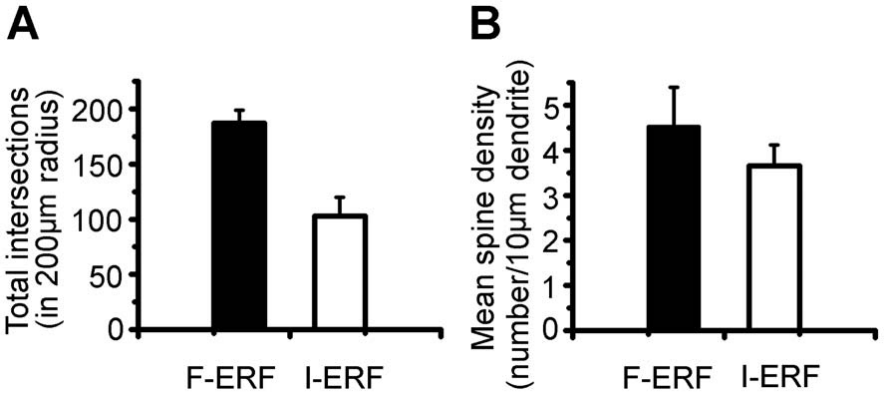
Total intersections and mean spine density of F-ERF and S-ERF neurons in same cortical layer. Four F-ERF neurons and five S-ERF neurons were analyzed, all were located in layer II/III. (A) Comparison in total number of intersections in 200 µm radius. The mean for the F-ERF neuron group is 187.0,
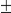
11.85 and that for the S-ERF neuron group is 103.2
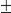
16.88. The difference is significant (P<0.05, t-test). (B) Comparison in mean spine density in 200 µm radius. The mean density for the F-ERF neuron group is 4.53
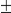
0.87, and that for the S-ERF neuron group is 3.66
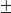
0.46. The difference is not significant (P>0.05, t-test). Error bars represent SEM. All values are corrected for shrinkage.

The above comparison in dendrite morphology between F-ERF and S-ERF cells was made for all cells in spite of their lamina location. Considering the heterogeneity of cells in different layers, in Figure 7 we compared the number of Sholl intersections and spine density only for those cells whose cell bodies were located within layer II/III and they were morphologically pyramidal cells. The data were based on analyses from 4 F-ERF cells (No. 1, 2, 3, 7 of Table 1) and 5 S-ERF cells (No. 22, 23, 24, 30, 31 of Table 1). Figure 7A illustrates the difference in total number of intersections in the range of 10 Sholl circles (up to 200 µm from the soma), the results show that the number of intersections for the F-ERF neurons (mean 187.0,
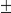
11.9,SEM) is significantly more than that of the S-ERF neurons (mean 103.2,
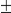
16.9, SEM). Figure 7B compares spine density (number of spines/10 µm dendrite) between the two types of cells, higher spine density was found for the F-ERF cells (mean 4.53, 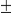
0.87, SEM) than for the S-ERF cells (mean 3.66, 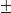
0.46, SEM), but the difference is not significant (*p*>0.05).

### Comparison of axonal morphology between F-ERF and S-ERF neurons

**Figure 8:**
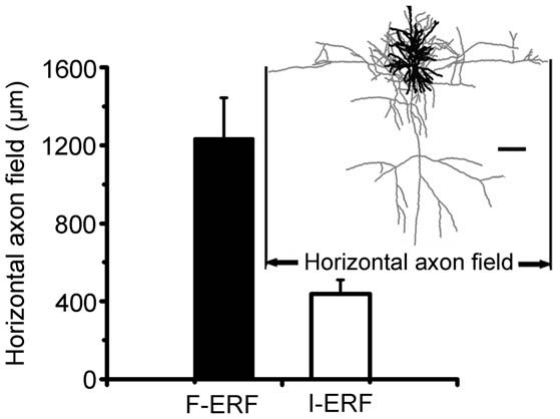
Histograms showing a comparison of horizontal width of axon field between F-ERF and S-ERF cells. The horizontal width of axon field (shown by thin lines in inset) is defined as the maximal distance between the utmost tips of the filled axons in horizontal focal planes. Height of the columns indicates the mean width of axon field obtained from 9 F-ERF cells and 10 S-ERF cells. Error bar indicates SEM. The difference is significant between the two types of neurons (P<0.01, t-test). Horizontal bar = 100 µm. The values are corrected for shrinkage.

The distinction in axon morphology between F-ERF and S-ERF cells is also apparent. The axons of most F-ERF cells form a plexus of long-range connections running parallel to the cortical layers, and these collaterals may distribute widely in the same layer in which the cell body is located and also in other cortical layers. In Figure 2, for example, the axon collaterals of all the seven F-ERF cells (No. 1–4, 10, 12, 17) distributed vertically across several cortical layers and expanded horizontally over a wide range. The longest span of an axon field in our sample was 2.5mm (cell No. 2). In contrast, for all of the S-ERF cells illustrated in Figure 2, the axon branches were close to the cell body and the collaterals were more restricted, some were in the same cortical layer (No. 22, 24,30,31) and others extends slightly into the neighboring layers. Figure 8 shows a comparison of the width of axon fields between F-ERF (n = 9) and S-ERF cells (n = 10). Horizontal axon fields of the F-ERF neurons varied between 693.0 µm and 2750.0 µm (mean 1233.9
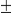
210.0 µm, SEM) and that of the S-ERF neurons were between 213.4 µm and 649.0 µm (except one with a 968.0 µm field width) (mean437.0
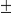
71.8 µm, SEM). The difference is significant (P<0.01, t-test).

### Difference in soma size between F-ERF and S-ERF neurons

**Figure 9:**
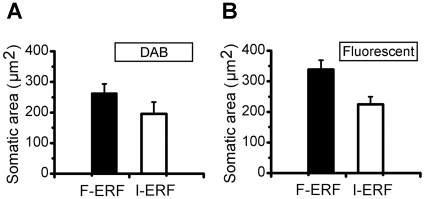
Comparisons of soma area values between F-ERF and S-ERF neurons. (A) Comparison based on DAB-identified neuron. The mean somatic area for the F-ERF neuron group (n = 5) is 263.4
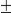
30.1 µm^2^ and that for the S-ERF neuron group (n = 5) is 196.0
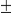
38.4 µm^2^. The values are corrected for shrinkage. (B) Comparison based on fluorescent dye (streptavidin-Texas Red.) identified neurons. The mean somatic area for the F-ERF neuron group (n = 6) is 339.0
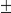
30.2 µm^2^ and that for the S-ERF neuron group (n = 7) is 224.7
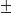
25.2 µm^2^. The difference in soma area between the two types of neurons is significant (P<0.05, t-test) both in (A) and (B). Error bars represent SEM.

Although morphologically both the F-ERF and S-ERF neurons were mostly pyramidal cells, a comparison of soma areas between the two types of pyramidal cells shows a significant difference. The results are shown in Figure 9. As fluorescent dye image shows less tissue shrinkage than that treated by DAB during histological processing, we compared the soma size separately in two groups of data. The measurements shown in Figure 9A were obtained from the DAB identified cells. The soma areas of the F-ERF pyramidal neurons in all the layers ranged from 233.5 to 314.0 µm^2^ with a mean of 263.4 µm^2^(SEM = 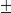
30.1 µm^2^, n = 5) and that of the S-ERF pyramidal neurons ranged from 147.0 to 247.7 µm^2^ with a mean of 196.0 µm^2^ (SEM = 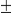
38.4 µm^2^, n = 5) (P<0.05, t-test). Similar differences were found in the group identified with fluorescent dye (Figure 9B) where the mean area of the F-ERF cells is 339.0 µm^2^ (ranged from 237.6 to 445.7 µm^2^, SEM = 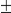
30.2 µm^2^, n = 6,), and that of the S-ERF cells is 224.7 µm^2^ (ranged from 123.5 to 327.2 µm^2^, SEM = 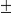
25.2 µm^2^n = 7) (P<0.05, t-test). It is clear that, in both groups, the soma area of F-ERF neurons is significantly larger than that of S-ERF neurons.

## Discussion

Evidence has been provided in a number of studies that the extensive region beyond the CRF, although alone unresponsive to visual stimuli,can exert robust suppressive or facilitative effects on the response to the stimuli in the CRF [4], [7], [9], [21], [32]–[34]. These two types of spatial summation have been assumed to play different roles in visual information processing. Facilitatory surround effects were assumed to involve in contour integration [34], [35] and in detection of broad field homogeneous textures [6]; suppressive surround effects were supposed to contribute to heterogeneity detection of local visual features, such as local discontinuity in orientation [5], [36], in spatial frequency, in color, speed and direction of movement [37], in relative spatial phase [38], and in relative moving speed [10], and viewed as the basis for perceptual ‘pop-out’ and illusory contours [21], [23], [34], [39], [40]. Despite the intensive researches, the existence of facilitative surround effect has remained a matter of controversy. Some authors regard the length summation area as part of the receptive field center and conclude that there are no facilitatory inputs from the surround [3], [41]. Others have demonstrated that facilitatory effects can be elicited by presenting discrete stimuli over regions beyond the length summation area [34]. Recent studies emphasize the dynamic nature of the center-surround interactions based on the results that the length summation areas of cortical neuron receptive fields are not fixed, but vary as a function of stimulus contrast [35], [42], [43]. Thus, it is not clear yet whether the neurons showing suppressive surround effect are morphologically different from those showing facilitatory surround effect. The aim of the present study was to answer the question and to establish morphological bases for the facilitatory/suppressive classification of surround modulation. In this study, we selected exclusively the cortical neurons that showed robust facilitative surround effect (*SI* 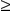
0.2) or suppressive surround effect (*SI* 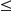
−0.4) under a medium contrast level (contrast = 0.4). We have investigated a total of 44 V1 cells for which both area-summation properties and intracellular staining were obtained. From the sample, about 90.5% (19/21) of the F-ERF neurons and 82.6% (19/23) of the S-ERF neurons had the morphology identifying them as pyramidal cells, only 2 out of 21 F-ERF cells and 4 out of 23 S-ERF cells were non-pyramidal cells. As pyramidal cells are heterogeneous in relation to soma size, axon distribution, spine size and density [44], [45], we compared the measurements of these morphological characteristics between the pyramidal cells exhibiting different ERF modulation (F-ERF, I-ERF). The main findings were: (1) the soma area of F-ERF pyramidal neurons are significantly larger than those of S-ERF pyramidal neurons (Figure 9), (2) F-ERF pyramidal neurons have more complex dendritic arborization and a higher spine density than S-ERF neurons (Figure 3 and Figure 7), (3) statistically,the heads of dendrite spines are larger in the F-ERF cells than S-ERF cells (Figures 4–6), (4) axons of most F-ERF cells form a plexus of long-range connections expanded horizontally over a wide range (mean 1233.9
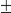
210.0 µm, SEM) and these may distribute vertically across several cortical layers, in contrast to the situation with regard to the axon collaterals of S-ERF cells which are restricted within a shorter range (mean437.0
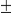
71.8 µm, SEM) and distributed mainly within the same cortical layer (Figure 2 and Figure 8). These morphological differences in structure of dendrites and axons are important for the sampling and integrating characteristics of the two types of spatial summation properties.

The differences in spine density and size of spine heads between F-ERF and S-ERF cells are functionally important. Dendritic spines are known to be the recipient of most excitatory synapses to pyramidal cells [46]–[49], so that the number of dendritic spines provides a good estimate of the number of excitatory synapses that different types of pyramidal cells receive. Recent studies have reported that the spine head volume is correlated with the number of postsynaptic receptors [31], [49]–[53], and the number of pre-synaptic docked vesicles [49], [51], [52]. Therefore, the higher spine density and larger head volume of F-ERF neurons may correlate with more efficient excitatory synaptic transmission than that of S-ERF neurons.

Axons of cortical pyramidal neurons, which make long-range horizontal connections, are known to be the underlying mechanism for spatial integration of inputs from the extra-receptive field [55]–[57]. Earlier studies reported that the axon collaterals of some pyramidal cells extend horizontally for a long distance and form discrete, stripe-like clusters in the neighboring columns that share similar orientation preferences [57]–[63]. The present experiments demonstrated that only neurons with F-ERF properties possess such long-range horizontal connections, which explains the fact that the most effective stimuli for eliciting surround facilitation are broad field gratings presented at the cell's preferred orientation. On the contrary, the neurons showing S-ERF properties mostly possess short-range axon connections that prefer surround stimuli presented at the orthogonal orientations, and iso-orientation stimuli over center and surround produce suppression of neuron responses.

In conclusion, the above results demonstrate that although the vast majorities of both F-ERF neurons and S-ERF neurons are pyramidal cells, the F-ERF and S-ERF cells differ substantially in their soma sizes, complexity of dendritic branching, spine size and density and the extent of the cortical spread of their axons.

## Materials and Methods

### Ethics Statement

This study was carried out in strict accordance with the recommendations in the Guide for the Care and Use of Laboratory Animals of the National Institutes of Health. The protocol was specifically approved by the Committee on the Ethics of Animal Experiments of the Shanghai Institutes for Biological Sciences, Chinese Academy of Sciences (Permit Number: ER-SIBS-621001C). All surgery was performed under general anesthesia combined with local application of Lidocaine (for details see “animal preparation”), and all efforts were made to minimize suffering.

### Animal preparation

Acute experiments were performed on 21 adult cats. Detailed descriptions of procedures for animal surgery, anesthesia, and recording technique are available in [64]. Briefly, cats were anesthetized prior to surgery with ketamine hydrochloride (30 mg/kg i.m.), and then trachea and femoral vein were cannulated. After the operation, the animal was placed in a stereotaxic frame for craniotomy and subsequent visual experiments. Lidocaine was applied to all wound margins and pressure points. A craniotomy (2 mm diameter) was performed at the recording site in the striate cortex. During recording, anesthesia and paralysis were maintained with urethane (20 mg/kg/hr) and gallamine triethiodide (10 mg/kg/hr), respectively, and physiological stability with glucose (200 mg/kg/hr) in Ringer's solution (3 ml/kg/hr). End-expiratory CO_2_ was kept at 4% and core body temperature at 38°C. Electroencephalogram and ECG were monitored continuously. Anaesthesia was considered to be sufficient when the EEG reflected a permanent sleep-like state and the heart rate remained stable at an appropriate frequency. The nictitating membranes were retracted and pupils dilated with topic application of 5% neosynephrine and 1% atropine. Artificial pupils with a 3 mm diameter were used. Contact lenses and additional corrective lenses were applied to focus the retina onto a screen.

### Enzyme treatment and electrode preparation of the dura

To render the dura permeable to a patch electrode whilst maintaining its integrity, the exposed dura was treated with purified collagenase [65] by applying the enzyme (50 mg/ml) with a small piece of filter paper placed on the dura for 20–30 mins. Then the filter paper was removed and the area of application rinsed with physiological saline. The glass pipettes (Sutter Instrument Company, BF150-86-10) were pulled on a P-97 (Sutter Instrument Company, USA) microelectrode puller (tip diameter about 1 µm, resistance about 10 MΩ) and were filled with a solution buffered to pH 7.4 containing (in mM) KCl 90, NaCl 10, potassium EGTA 5 and HEPES buffer 10. For subsequent intracellular staining of a neuron, the solution also contained 1% biocytin. The micropipette was connected to the input of an intracellular recording amplifier (World Precision Instruments, USA). A hydraulic, pulse-motor driving unit (PP5-1, Narishige, Japan) was used to advance or retract the electrode. Prior to penetrating the dura, the hole in the skull was filled with 4% agar dissolved in saline, to attenuate cortical pulsations. Successful penetration of the electrode through the dura was confirmed by monitoring the change in tip resistance during an applied pulse of electrical current (100 µs, 0.1 nA, interval 500 ms), compared to the original resistance tested in the agar. To prevent the electrode tip from becoming blocked, a positive pressure of 20 kPa was applied while advancing the electrode. When the electrode tip touched the surface of the dura, both the tip resistance and the baseline noise would increase 2–3 times compared to the reference value. This increase in the tip resistance persisted until the electrode pierced the dura. A final decline in tip resistance (to the reference value tested in the agar) and the baseline noise level, coupled with the appearance of neuronal discharges, indicated the tip's successful entry into the cortical tissue. At this point, the positive pressure applied to the electrode was reduced to 3–5 kPa.

### Visual Stimuli

A personal computer (Intel-P4 CPU, 2.0 GHz, Memory 1G) with a graphics card (NVIDIA GeForce 6200) was used to generate visual stimuli on the monitor (frame rate, 100 Hz). The screen was 40×30 cm. This visual stimulator could generate multiple patches of sinusoidal grating stimuli. Under computer control, the grating orientation, spatial and temporal frequency, and movement direction were matched to the preferred parameters of the cell under study and real-time analyses of the responses were performed. The monitor was placed 57 cm from the eyes. The contrast of the gratings was 40% and mean luminance, 10 cd/m^2^. All measurements were made during stimulation of the neuron's dominant eye with the other eye occluded. All cells recorded were obtained from the area of the cortex representing the central 10° (radius) of the visual field.

### Intracellular injection

The electrode pipette was advanced slowly into the cortex to search for visually responsive cells while maintaining a positive pressure (3–5 kPa). When the large action potentials changed from being bipolar to unipolar, the positive pressure was reduced to 1.0–2.0 kPa, and the resistance was continuously monitored with current pulses (0.1 nA, 10 ms, 1 pulse/s). Close contact with a neuron is recognized by an increase in the resistance of the electrode. At this point, the positive pressure was released and a small negative pressure was applied (1.0–2.0 kPa). This often results in gradual penetration of the cell interior, as is indicated by a slow increase in membrane potential. When the membrane potential became stable, at a value lying between –40 and –60 mV, the negative pressure was released, in order to avoid sucking in the intracellular contents. On average, the state suitable for intracellular recording was usually maintained for more than 1 hour, which was ended by intracellular injection of biocytin [27] to investigate the morphological characteristics of the recorded cell. The injection was performed by passing negative current pulses, 1.0 nA in a 100 ms on/200 ms off cycle, for a period about 10 min. During current injection, the normal responsiveness of the cell was continuously monitored using a visual stimulus. Then the electrode was withdrawn slowly from the cell interior and the responsiveness of the cell was checked again extracellularly. For the next penetration, a new electrode was used and the recording site was placed at least 1 mm away from the previous penetration. One experiment normally lasted 3 days; and two to three cells could be intracellularly labeled and identified after completion the experiment.

### Histological procedures

At the end of the experiment, the animal was deeply anaesthetized and perfused through the heart, first with 0.9% saline and then with 4% paraformaldehyde in phosphate buffer (PB), pH 7.4. Tissue blocks containing the injected cells were removed and post-fixed overnight at 4°C in the same fixative solution and stored in 0.1 M PB. The tissue was serially sectioned in the coronal plane on a vibratome at a thickness of 80 µm. The sections were thoroughly washed in PBS followed by Tris-buffered saline (TBS), and were pretreated for 6 h in a 0.5% solution of Triton X 100 in PBS. Injected cells were identified by incubating the tissue overnight in the avidin-biotin HRP complex at 4°C in dilution 1∶2500 in PBS. The enzyme reaction was revealed with diaminobenzidine (DAB, 0.06%) and H_2_O_2_ (0.003%) in 0.01M PBS (40°C) for 15 min. The sections were thoroughly rinsed and mounted on gelatin-coated slides. The layer localization of the injected cells was determined with Nissel-staining method. Only neurons that had no obvious truncation of their dendritic and axonal profiles were used for quantitative morphology analysis.

### Quantitative analysis of cell morphology

The outline of neurons revealed with DAB was drawn by a camera lucida attached to NIKON E600FN microscope with a 60× oil objective (1.4 NA). For some neurons, three-dimensional reconstructions were made using the Neurolucida software (Microbrightfield, Inc, USA). For further quantitative analysis, the following morphological parameters were measured: (1) number of bifurcations of dendrites branching determined by Sholl analysis [30], (2) areas of the cell body, (3) spine density (number of spines/10 µm) of dendrites, (4) the maximal horizontal field span of the axons. For measuring the maximal span of the axons, two-dimensional projections of the axon trees were traced on a digitizing tablet and axon terminals were registered from 10–32 adjoining sections. Alignment of neighboring sections was carried out with the help of corresponding cut ends of labelled axonal processes in the estimated axon projection field of the labeled cells.

Images of the neurons shown by DAB were also examined under the confocal microscope (LSM510, Zeiss, Germany) with an Aprochromat 60× oil objective setting at 1024×1024 pixel resolution. Each image was a z-series projection of several images that averaged two to three times and taken at 0.5–1 µm depth intervals. Area of spine head were measured automatically using image analyzing software (LSM510, Zeiss, Germany). The spine head measurements were limited to the thin and mushroom-shaped spines, as the head of stubby spines was difficult to distinguish from the dendrite shaft. For each neuron, ten dendrite segments were examined and twenty spines were sampled for each segment. A total of 1600 spines in 80 dendrite segments (60 to 80 µm) were sampled. Scaling spines started from the embranchment point, and measured one by one along the dendrite tree.

Some injected cells were identified with fluorescent dye (streptavidin-Texas Red, Vector labotatories, USA, 1∶1000) in a 0.3% solution of Triton X 100 in 0.01 M PBS at 4°C overnight. The labeled neurons were examined with the same confocal microscope and reconstructed using the affiliated image analysis software. The somatic area of fluorescent identified neurons was measured with EeuroExplorer software (Microbrightfield, Inc, USA) by drawing the outline of the cell body with Neurolucida software. The statistical significance of the experimental data was tested using a Student's t-test.

### Correction for shrinkage

To estimate the shrinkage during histological procedure, we measured the sizes of the sections before and after the histologic procedures at the horizontal (x/y) plane under microscopic observation. The shrinkage thus calculated was 10.4±1.6% (SEM, n = 15). The shrinkage along the z-axis was estimated by comparing the thickness of sections as they come off the microtome and after histological processing with Neurolucida. The averaged shrinkage thus measured was 47.8±1.2% (SEM, n = 15). The greater shrinkage in the z-plane is probably due to the fact that the deformation was mostly restricted to the z-axis while vibratome sections were dried on glass slide [66].
